# SHP2 in TAMs promoted the survival of gastric adenocarcinoma via suppressing the P38/ERK1/2/SP1/BRD4/STING induced inflammation and ROS

**DOI:** 10.3389/fmed.2026.1789222

**Published:** 2026-04-30

**Authors:** Jing Bai, Hui Ren, Yachen Zhang, Xiao Li

**Affiliations:** 1 Medical Records Department, Xingtai People’s Hospital, Xingtai People's Hospital, Xingtai, China; 2 Department of Pharmacy, Xingtai People's Hospital, Xingtai, China; 3 Department of Traditional Chinese Medicine, Xingtai People's Hospital, Xingtai, China

**Keywords:** gastric adenocarcinoma, inflammation, ROS, SHP2, TAMs

## Abstract

**Background:**

It is well established that tumor-associated macrophages (TAMs) are crucial to the development of tumors. Here, we looked into how SHP2 and M2/M1 macrophages affected gastric cancer.

**Methods:**

Bulk RNA-seq analysis was conducted using the GSE118916 dataset from the GEO database to screen for differentially expressed genes, and GO, KEGG enrichment analysis, and immune cell infiltration correlation analysis were performed. We also used PMA to differentiate THP-1 cells (M0 macrophages). To simulate the association between gastric adenocarcinoma and TAMS, M0 macrophages were co-cultured with AGS (human gastric adenocarcinoma cells). To mimic the advanced stage of gastric adenocarcinoma the co-cultured cells were cultured in 1% O2 low serum and low sugar. SHP2 overexpression, SHP2 knockdown, and SP1 inhibitor BDR4 inhibitor JQ-1 were used to examine the effects of SHP2, SP1, and BRD4 on macrophage polarization and cancer cell death, migration, and invasion.

**Results:**

Bulk RNA-seq analysis revealed that differentially expressed genes in gastric cancer were mainly enriched in extracellular matrix organization and adhesion-related pathways. Macrophages showed significant positive correlation with activated dendritic cells in the immune infiltration analysis. SHP2 overexpression inhibited the expression of p-P38, p-ERK1/2, p-SP1, BRD4, FOXM1, STING, NRLP3, and inflammation- and ROS-related cytokines IL-1β, TNFα, and MDA, and SOD, and the expression of M2 polarization-associated proteins, Arg-1 and Cathepsin K. The aforementioned proteins’ expression was greatly enhanced by SHP2. the aforementioned proteins’ expression. P-SP1 was significantly inhibited under the action of SP1 inhibitor, in addition, STING, NRLP3 and ROS-related proteins IL-1β, TNFα and MDA, SOD expression, M2 polarization-related proteins Arg-1, Cathepsin K. The expression of the aforementioned proteins was markedly decreased by the combination of SP1 inhibitor and BRD4 inhibitor. Additionally, there was an increase in cancer cell invasion, migration, and death.

**Conclusion:**

SHP2 in TAMs promotes gastric adenocarcinoma survival by inhibiting P38/ erk1 /SP1/BRD4/STING-induced inflammation and ROS.

## 1 Introduction

Gastric Adenocarcinoma (GA) is one of the most common malignant tumors worldwide, causing hundreds of thousands of deaths each year (1). Patients with advanced and metastatic illness still have poor survival rates, despite recent improvements in early detection and therapy (2). Tumor cells, fibroblasts, immune cells, and other various cell populations make up the tumor microenvironment (TME), a complex ecosystem that actively contributes to the growth and spread of tumors (3, 4). The most prevalent immune cells in the TME are called tumor-associated macrophages, or TAMs. And they are involved in various aspects of tumor immune escape, angiogenesis, invasion and metastasis through interactions with tumor cells, thus playing an important role in tumor development and progression (5).

Research on TAMs in various cancer types has gradually increased in recent years; of particular interest are the functions of macrophages of both M1 and M2 phenotypes in the tumor microenvironment (6, 7). Typically anti-tumor, M1-type macrophages may release pro-inflammatory cytokines (such as IL-1beta, IFNγ, and TNFα) and reactive oxygen species (ROS), boost immunity, eradicate infections and cells, and stop the formation of tumors (5, 8). M2-type macrophages, on the other hand, inhibit the immune response by secreting anti-inflammatory cytokines (such IL-10), encouraging angiogenesis and tissue remodeling, which in turn encourages the growth and spread of tumors (9, 10). The dynamic balance between these two phenotypes plays a key role in the tumor microenvironment, and many studies have been devoted to inhibiting tumor progression by modulating the polarization state of macrophages (11, 12).

SHP2 (Src homology 2 domain-containing protein tyrosine phosphatase 2), a member of the PTP family, is a crucial regulator of several cell signaling pathways (13). SHP2 may control a number of signaling pathways, including RAS-MAPK, PI3K-AKT, and JAK–STAT, which has an impact on cell survival, proliferation, and apoptosis (14). SHP2 has been demonstrated to be overexpressed in a number of cancer types and to be strongly linked to the malignant development and poor prognosis of malignancies. For example, in breast and lung cancers, SHP2 overexpression is associated with tumor aggressiveness and drug resistance. The biological function of SHP2 is significantly dependent on the cellular environment. By dephosphorylating negative regulatory molecules like RasGAP, SHP2 stimulates the RAS/MAPK/ERK signaling cascade in tumor cells, promoting cell survival and proliferation (15). In immune cells, the role of SHP2 is completely different. When recruited by inhibitory receptors, SHP2 exerts a signaling braking function by dephosphorylating proximal activating signaling molecules (16–18).

However, relatively few studies have been conducted on the specific role of SHP2 in TAMs and its effect on gastric adenocarcinoma. The question of whether SHP2 in TAMs affects gastric adenocarcinoma progression by regulating specific signaling pathways still needs to be further explored. A transcription factor called SP1 (Specificity Protein 1) binds to particular DNA sequences to control the expression of several genes, such as BRD4 (Bromodomain-containing protein 4) and STING (Stimulator of Interferon Genes), among others (19, 20). BRD4 is an epigenetic regulatory protein that promotes gene transcription by recognizing acetylated histones (21). However, STING is implicated in DNA recognition, interferon production, and the innate immune response (22). Thus, the purpose of this work was to examine the expression of SHP2 in TAMs and its impact on the ability of gastric cancer cells to migrate, undergo apoptosis, and invade. To study the role of SHP2 in TAMs, we co-cultured human gastric adenocarcinoma cell line AGS with PMA-differentiated THP-1 cells that imitate M0 macrophages. In addition, we explored the mechanism by which SHP2 promotes the survival of gastric adenocarcinoma cells by inhibiting the P38/ERK1/2/SP1/BRD4/STING signaling pathway and its associated inflammatory response and oxidative stress.

## 2 Methodology

### 2.1 Screening of differentially expressed genes in gastric adenocarcinoma and their biological significance based on the GSE118916 dataset

The gastric adenocarcinoma dataset GSE118916 was downloaded from the Gene Expression Omnibus (GEO). This dataset contains 15 samples of gastric adenocarcinoma (STAD) and 15 samples of normal tissues. In the data preprocessing stage, the normalizeBetweenArrays function from the limma package in R was used for data normalization to eliminate technical errors between batches. Differentially expressed genes (DEGs) between gastric cancer and normal samples were then found using differential expression analysis based on the DESeq2 method. The significance threshold was set as Adjusted *p*-value < 0.05 and |log₂ (multiple change)| > 1, and the *p*-values were corrected using the Benjamini-Hochberg method. The ClusterProfiler package was used to conduct GO and KEGG pathway enrichment analysis of the differentially expressed genes. The GO analysis included cellular components, molecular functions, and biological processes, while the KEGG analysis screened key biological pathways. The single-sample gene set enrichment analysis (ssGSEA) algorithm was used to calculate the standardized enrichment score (ssGSEA Score) for each sample based on the gene marker set of 28 immune cell subsets. Spearman correlation analysis was used to evaluate the correlation between differentially expressed genes.

### 2.2 Cell culture

Both AGS and THP-1 cell lines were cultured in RPMI-1640 medium supplemented with 10% fetal bovine serum (FBS) at 37 °C in a humidified atmosphere (5% CO2). THP-1 cells were treated with 100 ng mL-1 phorbol-12-myristate-13-acetate (PMA; Millipore, Shanghai, China) for 24 h to differentiate into macrophage-like cells. To establish a co-culture model of tumor-associated macrophages and gastric cancer cells, an indirect co-culture was performed using a 0.4 μm pore size Transwell chamber. THP-1 (10,000 cells per well) and AGS (4,000 cells per well) were, respectively, inoculated into 96-well plates containing 200 μL RPMI-1640 medium. THP-1 cells were inoculated into the upper chamber, and AGS cells were inoculated into the lower chamber. Three replicates were prepared for each group. The cultures were conducted for 24 h (37 °C, 95% humidity, 5% CO2) or were carried out in 96-well plates under hypoxic low serum low glucose conditions (1% O2, 1.0 g/L glucose, and 5% FBS). Cells were treated with Mithramycin A (MTA, 20 nmol/L), or JQ-1 (10 μM) after 24 h of incubation.

### 2.3 Transfection

Designed SHP2 shRNA mimics and inhibitors were synthesized by OBio (Shanghai, China). Targeted sequence: shSHP2: 5′-CGCTAAGAGAGAACTTAACTTTT-3′; Non-targeted control shRNA (shNC): 5′-CCTAAGGTTAAGTCGCCCTCG-3′. THP-1 cells were seeded in 6-well plates at a density of 2 × 10^5^ cells per well and cultured in RPMI-1640 medium containing 10% fetal bovine serum overnight. The next day, lentivirus particles were added, with an infection multiplicity (MOI) of 10 and 8 μg/mL polybrene was also added. After 24 h of infection, the fresh medium was replaced and 2 μg/mL puromycin was added for 7 days to establish a stable THP-1 cell line with reduced SHP2 expression. The knockdown efficiency was verified by Western blot.

### 2.4 Western blot

After the co-culture is completed, discard the culture medium and wash twice with PBS. Add 0.25% trypsin–EDTA to digest for 1–2 min. The AGS cells will detach from the walls first, while the macrophages remain adhered to the walls. Gently aspirate the detached AGS cells, and then collect the remaining macrophages after washing with PBS. Proteins were extracted using RIPA buffer (Sigma-Aldrich, United States) containing protease inhibitors. Protein concentration was determined by Bradford assay. 40 μg of protein was separated using 10% sodium dodecyl sulfate-polyacrylamide gel (SDS-PAGE) and subsequently transferred to a polyvinylidene difluoride membrane. The membrane was blocked with 5% skimmed milk powder in TBST solution for 1 h at room temperature. Incubate overnight with primary antibody at 4 °C using a solution against p-SHP2, p-P38, p-ERK1/2, p-SP1, BRD4, FOXM1, STING, NRLP3, Arg-1, Cathpsin K. The membranes were incubated with the primary antibody for 1 h at room temperature (Abcam, United Kingdom). The assay was incubated for 1 h at room temperature using peroxidase-coupled secondary antibody (CST, Sigma-Aldrich, United States) and determined by enhanced chemiluminescence (ECL; Thermo Fisher, United States).

### 2.5 Elisa

Quantification of interleukin-1beta (IL-1beta), tumor necrosis factor-alpha (TNF-alpha), superoxide dismutase (SDS), and other cell culture media was performed using an ELISA kit (Thermo Fisher Scientific, 88–7,391–22) according to the manufacturer’s instructions. Superoxide dismutase (SOD), malonaldehyde (MDA) levels. The optical density (OD) at 450 nm was measured using an enzyme marker (Thermo, United States).

### 2.6 Flow cytometry

To detect the apoptosis of AGS cells, the cells in the co-culture system were first separated according to the method described in Section 1.4. After removing the upper chamber, the AGS cells in the lower chamber were collected and washed twice with PBS. Annexin V-FITC Apoptosis Detection Kit (Vazyme, Nanjing, China) was used to detect apoptosis. Treated cells were collected and resuspended in 100 μL of 1 × binding buffer and incubated with 5 μL of membrane-associated protein V-fluorescein isothiocyanate (FITC) for 10 min at room temperature under dark conditions, and then incubated with 5 μL of propidium iodide (PI) for a further 10 min. The percentage of apoptotic cells was measured in a BD CytoFLEX flow cytometer.

### 2.7 Wound healing assay

With cell confluence at approximately 90%, scrape the monolayer with a 200 μL pipette and remove cell debris by washing with PBS. Add culture medium and incubate the plates at 37 °C. Wound healing was investigated at various time points. In addition, representative scrape lines were captured.

### 2.8 Transwell

Cells suspended in serum-free medium were inoculated in the upper chamber of the matrix gel (BD Biosciences) (Corning Life Science, Suzhou, China) and the lower chamber was filled completely with medium. The 6-well plates were placed in a 5% CO2 incubator at 37 °C for 48 h. Afterwards, the chambers were fixed with 4% paraformaldehyde and stained with crystal violet for microscopic observation.

### 2.9 Statistical analysis

Data were expressed as SD ± mean and statistically analyzed using Prism 7.0 (GraphPad, United States). Student’s two-tailed t-test or one-way ANOVA was used for comparisons, and *p* < 0.05 was considered statistically significant.

## 3 Results

### 3.1 Bulk RNA-seq analysis results

**Figure 1:**
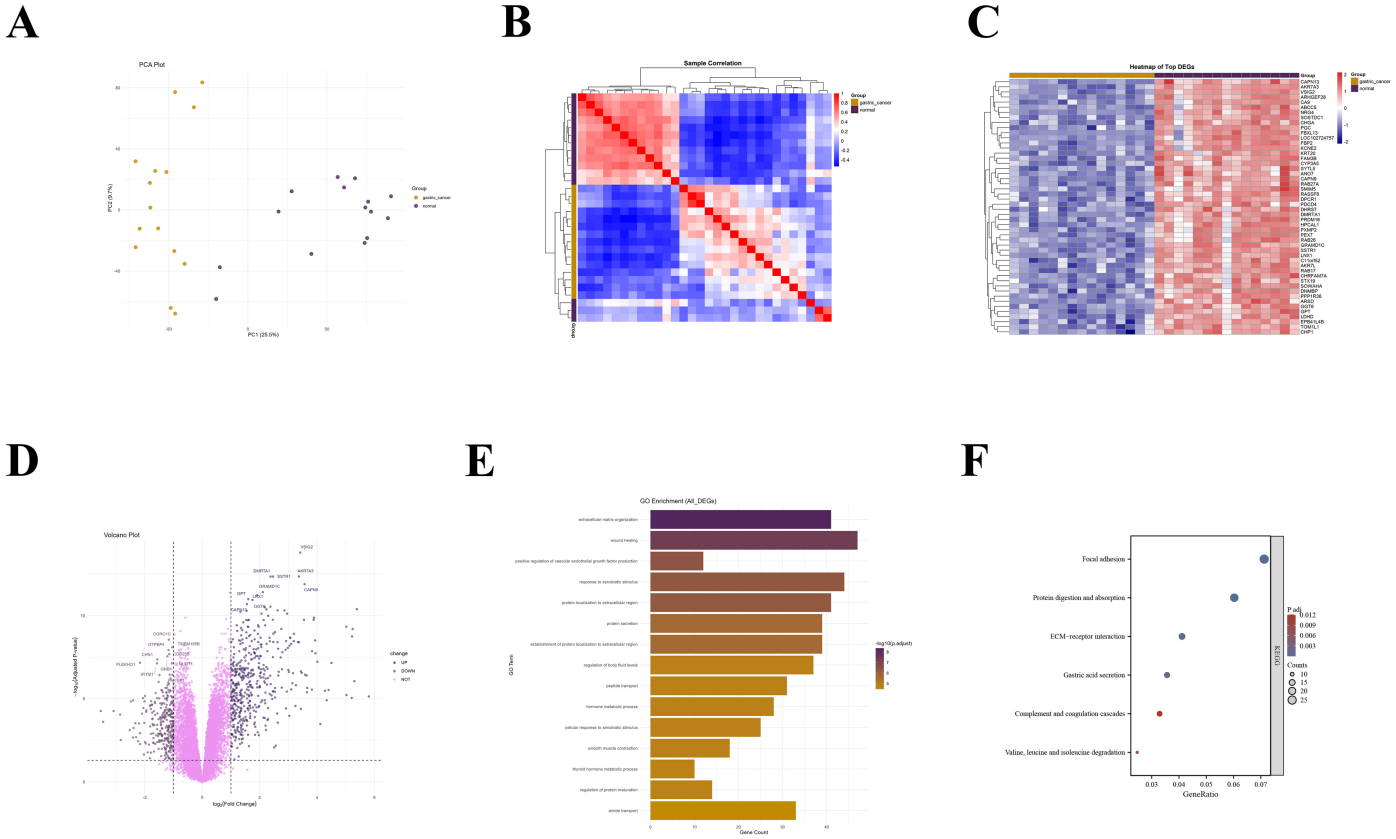
Single-cell transcriptome analysis of gastric adenocarcinoma reveals significant gene expression reprogramming. **(A)** PCA scatter plot of transcriptome data between gastric cancer group and normal group samples; **(B)** Heatmap of Pearson correlation coefficients between gastric cancer group and normal group samples; **(C)** Heatmap of differentially expressed genes; **(D)** Volcano plot of differentially expressed genes; **(E)** GO enrichment analysis graph; **(F)** KEGG enrichment analysis bubble plot.

**Figure 2:**
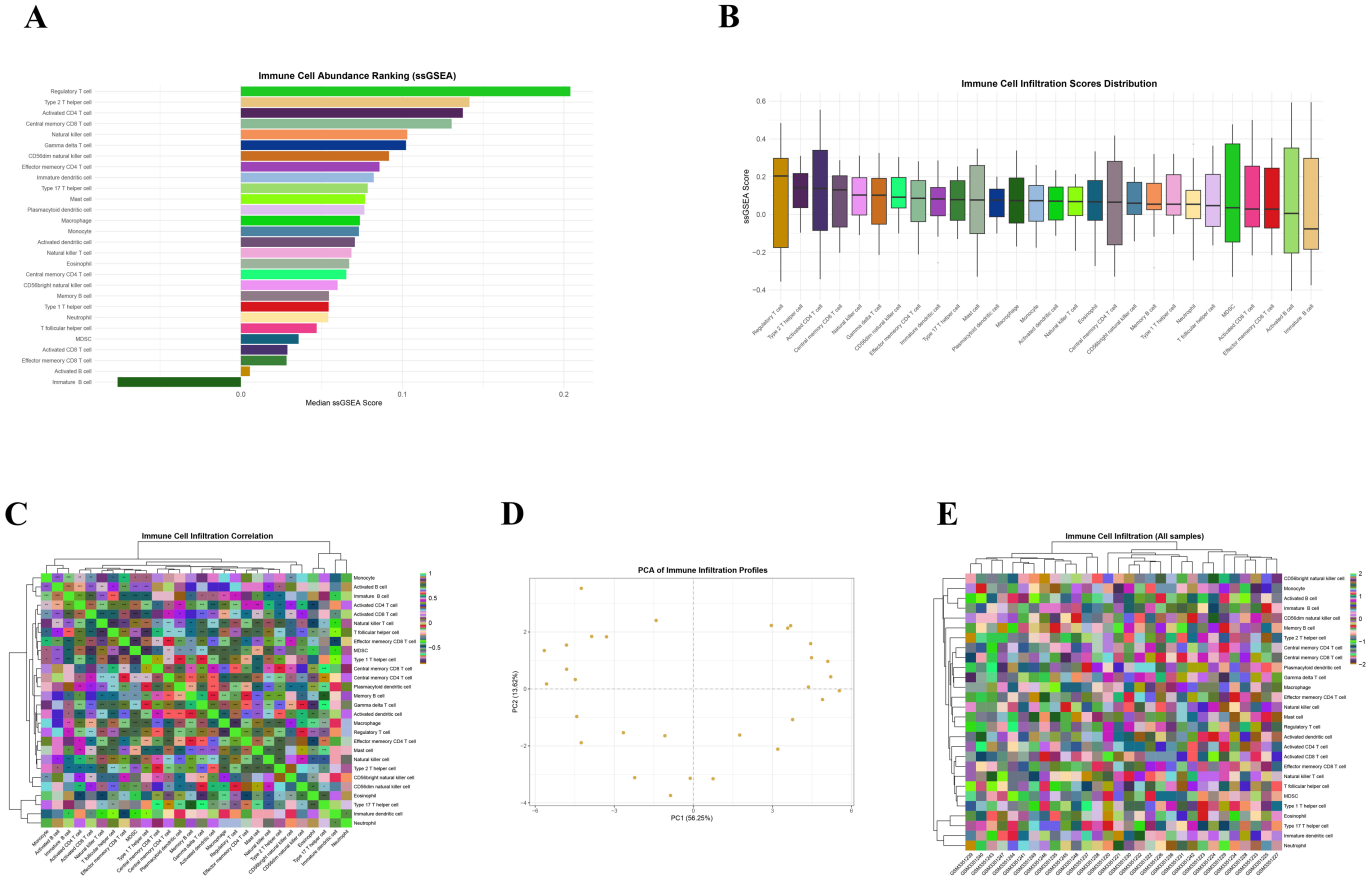
Immune landscape and functional pathway characteristics in the microenvironment of gastric adenocarcinoma. **(A)** Ranking diagram of immune cell abundance; **(B)** box plot of distribution of immune cell infiltration scores; **(C)** heatmap of correlation of immune cell infiltration; **(D)** PCA plot of immune infiltration characteristics; **(E)** heatmap of immune cell infiltration across the entire sample.

After analysis and screening using the limma package, a total of 745 significantly differentially expressed genes were identified in the gastric adenocarcinoma dataset, including 459 up-regulated genes and 286 down-regulated genes. PCA clearly demonstrated the systematic separation of gastric cancer tissues and normal tissues at the transcriptome level. The samples presented non-overlapping clustering patterns in the two-dimensional space formed by PC1 (accounting for 25.5% of the variance) and PC2 (accounting for 9.7% of the variance), indicating that the inter-group differences were significantly greater than the intra-group variations (Figure 1A). This separation feature was further confirmed in the sample correlation heatmap, where the Pearson correlation coefficients among intra-group samples were generally higher than 0.8, indicating good biological reproducibility, while the inter-group correlation coefficients were significantly reduced to below 0.2, highlighting the high specificity of the transcriptional profiles of the two groups of samples (Figure 1B). The heat map and volcano plot based on significantly differentially expressed genes (DEGs) showed that these genes could precisely distinguish gastric cancer from normal samples. Some genes such as VSIG2 and AKR7A3 showed a significantly high expression pattern in gastric cancer tissues, while genes such as CAPN13 and CHGA exhibited a clear down-regulation trend. This starkly different expression profile intuitively reflects the gene expression reprogramming during the gastric cancer development process (Figures 1C,D). GO analysis results showed that the differentially expressed genes were mainly involved in biological processes such as “extracellular matrix organization” and “wound healing,” with extremely high significance (Adjusted *p*-value < 1e-8), suggesting the active state of tissue remodeling in the tumor microenvironment. KEGG analysis further revealed this feature at the signaling pathway level, finding that the “ECM-receptor interaction” and “adhesion spot” pathways were significantly enriched, forming a perfect functional loop with the GO results (Figures 1E,F). ssGSEA analysis depicted a panoramic view of 28 immune cells in gastric cancer tissues. Regulatory T cells (Regulatory T cell), Th2 cells, and activated CD4 + T cells were the main components with the highest abundance in the microenvironment, while the abundance of neutrophils and Th17 cells was relatively low (Figure 2A). The heterogeneity analysis of samples showed that the infiltration levels of monocytes and activated B cells fluctuated greatly in different samples (Figure 2B). Correlation analysis revealed a complex cell interaction network, where activated dendritic cells and macrophages, as well as regulatory T cells, showed a significant positive correlation, suggesting that they may have played a synergistic role in constructing an immunosuppressive microenvironment (TME). Conversely, eosinophils were negatively correlated with multiple cytotoxic T cell subsets, suggesting different recruitment mechanisms or functional antagonism (Figure 2C). Based on immune characteristics, PCA analysis (PC1 explained 56.25% of the variance) and heat map clustering further confirmed that there were two distinct phenotypes between samples, “immune hyper-inflammation” and “immune tolerance,” reflecting the high complexity and heterogeneity of the gastric cancer immune microenvironment (Figures 2D,E). The results of the subsequent Spearman correlation analysis were provided in Supplementary file 1.

### 3.2 Effects of SHP2, SP1 and BDR4 on P38/ERK and P2X7R/FOXM1/STING/NLRP3 signaling pathways in THP-1 cells

**Figure 3:**
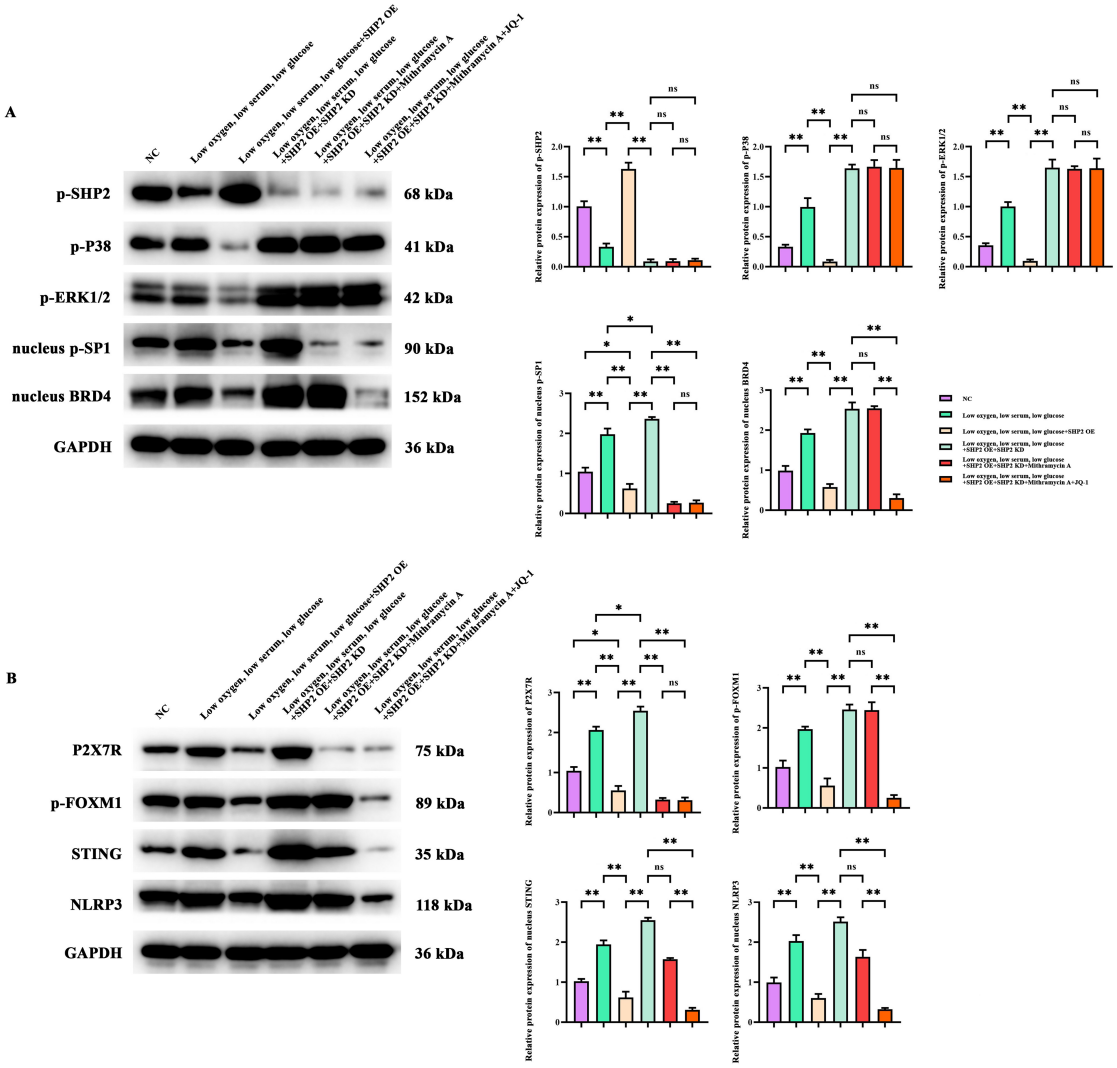
Effects of SHP2, SP1, and BDR4 on P38/ERK and P2X7R/FOXM1/STING/NLRP3 signaling pathways in THP-1 cells. **(A)** Western blot of p-SHP2, p-P38, p-ERK1/2, p-SP1, BRD4 in THP-1 cells. **(B)** Western blot of P2X7R, FOXM1, STING, NRLP3 in THP-1 cells. GAPDH/*β*-actin were used as the loading control. The data are presented as mean ± standard deviation. *N* = 3. *p* < 0.05 indicates statistically significant difference, *p < 0.05, **p < 0.01.

The Western Blot results showed that versus the control group, the protein expression of p-SHP2 in the Low oxygen, low serum, low glucose group was significantly attenuated, while the protein levels of p-P38, p-ERK1/2, p-SP1 and BRD4 were significantly increased; relative to the Low oxygen, low serum, low glucose group, the protein expression of p-SHP2 in the Low oxygen, low serum, low glucose + SHP2 OE group was elevated, while the protein expressions of p-P38, p-ERK1/2, p-SP1 and BRD4 were significantly reduced; in contrast to the control group, the protein expression of p-SP1 in the Low oxygen, low serum, low glucose + SHP2 OE group was also decreased; relative to the Low oxygen, low serum, low glucose + SHP2 OE group, the protein expression of p-SHP2 in the Low oxygen, low serum, low glucose + SHP2 OE + SHP2 KD group was significantly attenuated, while the protein expressions of p-P38, p-ERK1/2, p-SP1 and BRD4 were upregulated; in contrast to the Low oxygen, low serum, low glucose + SHP2 OE + SHP2 KD group, the protein levels of p-SHP2, p-P38, p-ERK1/2, p-SP1, BRD4 in the Low oxygen, low serum, low glucose + SHP2 OE + SHP2 KD + Mithramycin A group showed no significant difference, but the protein expression of p-SP1 was significantly reduced; in contrast to the Low oxygen, low serum, low glucose + SHP2 OE + SHP2 KD + Mithramycin A group, the protein expressions of p-SHP2, p-P38, p-ERK1/2 in the Low oxygen, low serum, low glucose + SHP2 OE + SHP2 KD + Mithramycin A + JQ-1 group showed no significant difference, but the protein expressions of p-SP1 and BRD4 were significantly decreased (Figure 3A).

The Western Blot results showed that compared with the control group, the protein expressions of P2X7R, FOXM1, STING, and NRLP3 in the Low oxygen, low serum, low glucose group were enhanced; versus the Low oxygen, low serum, low glucose group, the protein levels of P2X7R, FOXM1, STING, and NRLP3 in the Low oxygen, low serum, low glucose + SHP2 OE group were significantly attenuated, and when juxtaposed with the control group, the protein expression of P2X7R in the Low oxygen, low serum, low glucose + SHP2 OE group was also decreased; when juxtaposed with the Low oxygen, low serum, low glucose + SHP2 OE group, the protein levels of P2X7R, FOXM1, STING, and NRLP3 in the Low oxygen, low serum, low glucose + SHP2 OE + SHP2 KD group were heightened, and when juxtaposed with the Low oxygen, low serum, low glucose group, the protein expression of P2X7R in the Low oxygen, low serum, low glucose + SHP2 OE + SHP2 KD group was also increased; compared with the Low oxygen, low serum, low glucose + SHP2 OE + SHP2 KD group, the protein levels of FOXM1, STING, and NRLP3 in the Low oxygen, low serum, low glucose + SHP2 OE + SHP2 KD + Mithramycin A group showed no significant difference, but the protein expression of P2X7R was significantly decreased; compared with the Low oxygen, low serum, low glucose + SHP2 OE + SHP2 KD + Mithramycin A group, the protein levels of FOXM1, STING, and NRLP3 in the Low oxygen, low serum, low glucose + SHP2 OE + SHP2 KD + Mithramycin A + JQ-1 group were significantly diminished, while the protein expression of P2X7R showed no significant difference, and compared with the Low oxygen, low serum, low glucose + SHP2 OE + SHP2 KD group, the protein expressions of P2X7R, FOXM1, STING, and NRLP3 were significantly downregulated (Figure 3B).

### 3.3 Effects of SHP2, SP1 and BDR4 on ROS and inflammatory responses and AGS cell apoptosis in THP-1 cells

**Figure 4:**
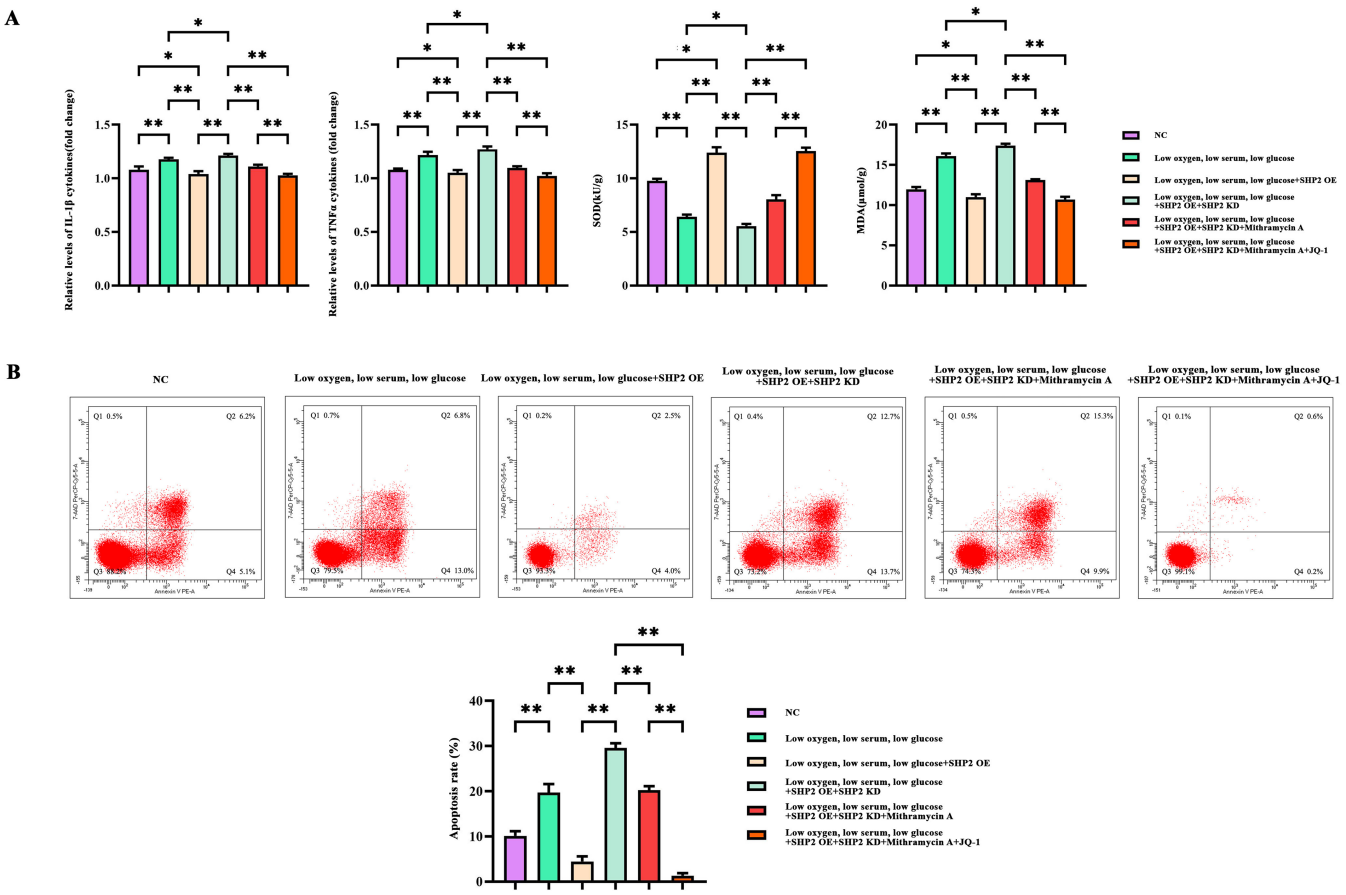
Effects of SHP2, SP1, and BDR4 on ROS and inflammatory responses and AGS apoptosis in THP-1 cells. **(A)** ELISA of IL-1β, TNFα, SOD, and MDA. **(B)** Flow cytometry detection of AGS cell apoptosis. The data are presented as mean ± standard deviation. *N* = 3. *p* < 0.05 indicates statistically significant difference, *p < 0.05, **p < 0.01.

The levels of pro-inflammatory cytokines IL-1Bata, TNFα, and MDA were significantly higher in the hypoxic low serum low glucose group than in the NC group, and the levels of SOD were reduced. MDA is a biomarker of lipid peroxidation and a key product of oxidative stress, whereas SOD and GSH are endogenous antioxidants that protect cells from oxidative damage. The levels of IL-1Bata, TNFα, and MDA were decreased and the level of SOD was increased by SHP2 OE. The levels of IL-1Bata, TNFα, and MDA were increased and the level of SOD was decreased by SHP KD. The levels of Mithramycin were significantly higher than those of NC group. IL-1Bata, TNFα, MDA levels were reduced and SOD levels were increased after treatment with Mithramycin A. IL-1Bata, TNFα, MDA levels were significantly reduced and SOD levels were heightened after JQ-1 treatment (Figure 4A).

We further examined the effects of SHP2, SP1 and BDR4 in apoptosis. Hypoxia low serum low glucose induced apoptosis in tumor cells, SHP2 OE inhibited apoptosis, and SHP2 KD reversed the effect of SHP2 OE. mithramycin A inhibited SHP2 KD tumor cell apoptosis, and JQ-1 significantly inhibited SHP2 KD tumor cell apoptosis (Figure 4B).

### 3.4 Effects of SHP2, SP1 and BDR4 on M2 polarization and AGS cell migration invasion in THP-1 cells

**Figure 5:**
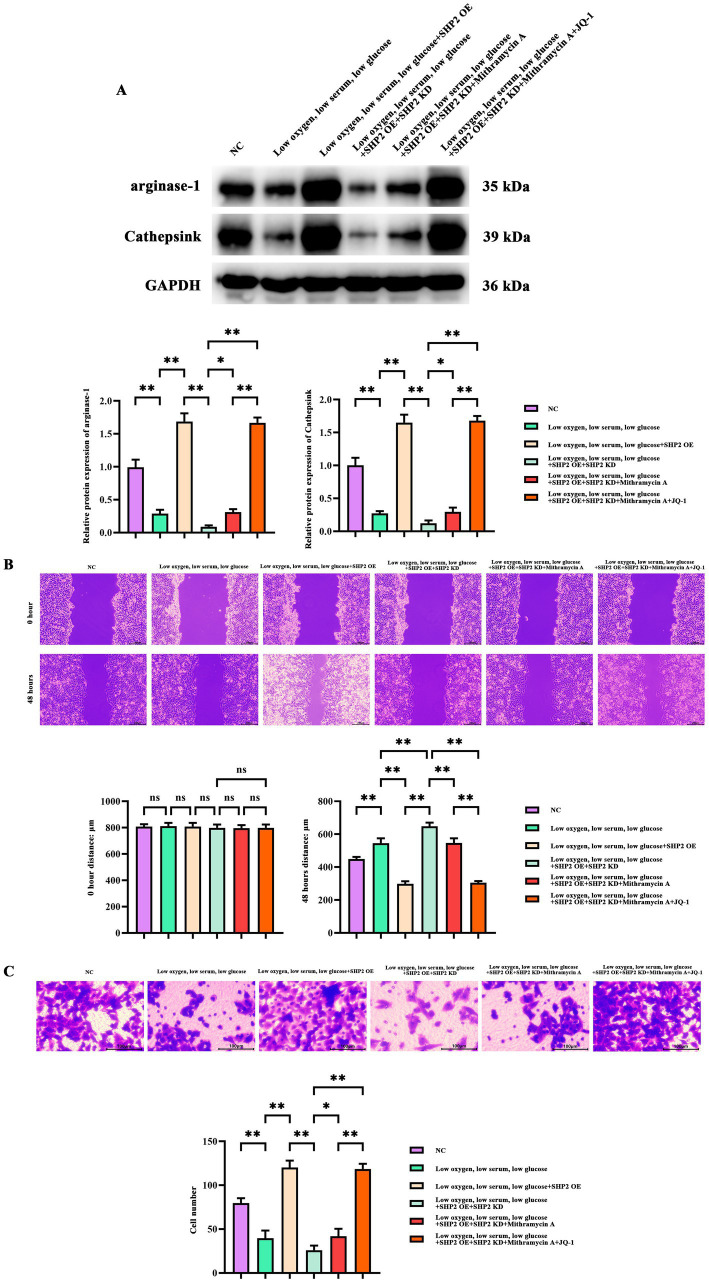
Effects of SHP2, SP1, and BDR4 on M2 polarization of THP-1 cells and AGS cell migration invasion. **(A)** Western blot detection of Arginase-1, Cathpsin K expression. GAPDH/β-actin were used as the loading control. **(B)** Wound healing assay to detect AGS cell migration ability. **(C)** Transwell detection of AGS cell invasion ability of AGS cells. The data are presented as mean ± standard deviation. *N* = 3. *p* < 0.05 indicates statistically significant difference, *p < 0.05, **p < 0.01.

The Western Blot results showed that compared with the control group, the expressions of THP-1 M2 polarization-related proteins Arg-1 and Cathepsin K in the Low oxygen, low serum, low glucose group were significantly attenuated; compared with the Low oxygen, low serum, low glucose group, the expressions of THP-1 M2 polarization-related proteins Arg-1 and Cathepsin K in the Low oxygen, low serum, low glucose + SHP2 OE group were upregulated; when juxtaposed with the Low oxygen, low serum, low glucose + SHP2 OE group, the expressions of THP-1 M2 polarization-related proteins Arg-1 and Cathepsin K in the Low oxygen, low serum, low glucose + SHP2 OE + SHP2 KD group were significantly reduced; versus the Low oxygen, low serum, low glucose + SHP2 OE + SHP2 KD group, the expressions of THP-1 M2 polarization-related proteins Arg-1 and Cathepsin K in the Low oxygen, low serum, low glucose + SHP2 OE + SHP2 KD + Mithramycin A group were increased; in contrast to the Low oxygen, low serum, low glucose + SHP2 OE + SHP2 KD group, the expressions of THP-1 M2 polarization-related proteins Arg-1 and Cathepsin K in the Low oxygen, low serum, low glucose + SHP2 OE + SHP2 KD + Mithramycin A + JQ-1 group were upregulated, and compared with the Low oxygen, low serum, low glucose + SHP2 OE + SHP2 KD + Mithramycin group, the expressions of THP-1 M2 polarization-related proteins Arg-1 and Cathepsin K in the Low oxygen, low serum, low glucose + SHP2 OE + SHP2 KD + Mithramycin A + JQ-1 group were heightened (Figure 5A).

**Figure 6:**
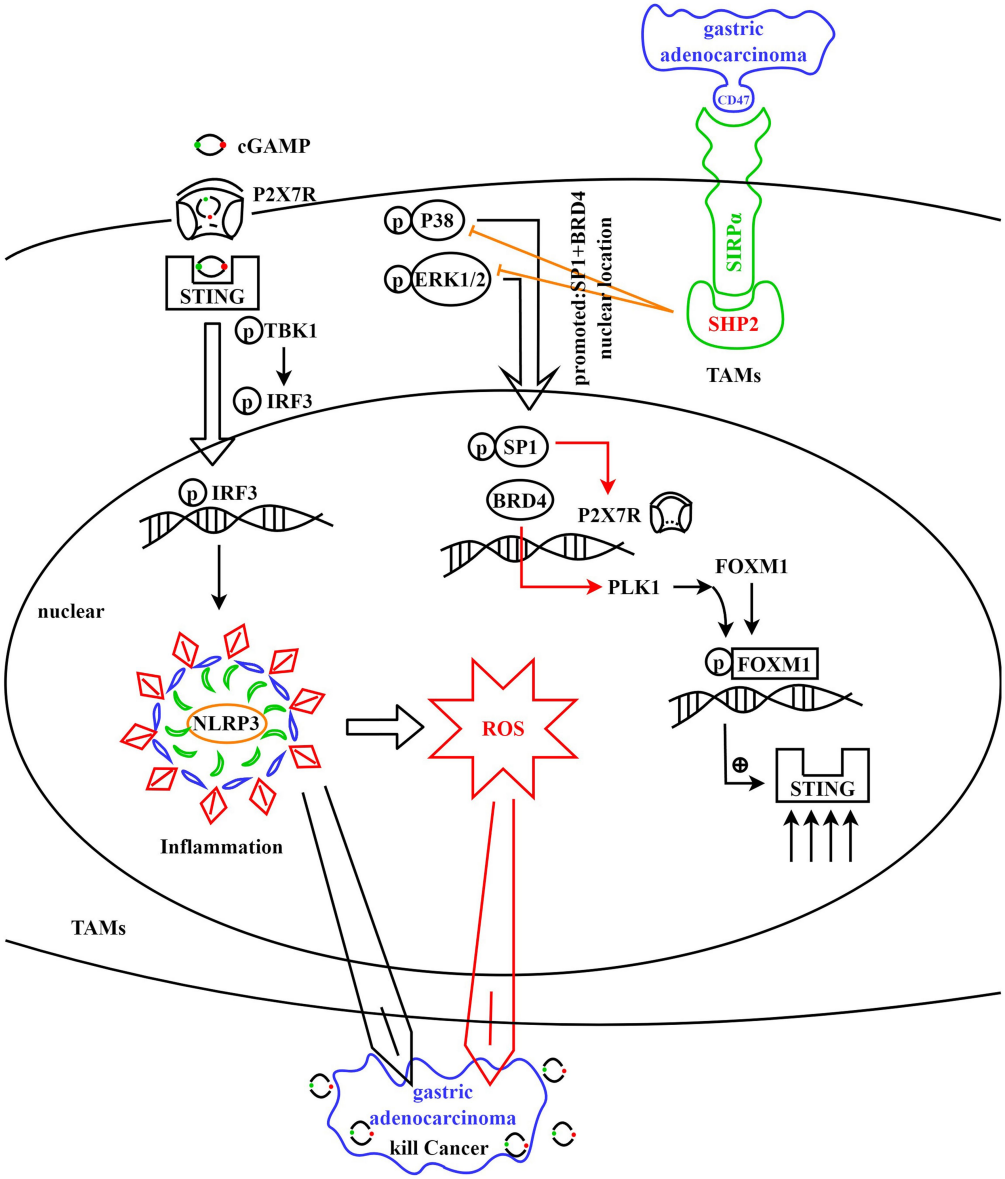
SHP2 in TAMs promoted the survival of gastric adenocarcinoma via suppressing the P38/ERK1/2/SP1/BRD4/STING induced inflammation and ROS.

We further examined the effects of SHP2, SP1 and BDR4 in cell migration and invasion. Hypoxia low serum low glucose inhibited tumor cell migration and invasion, SHP2 OE promoted cell migration and invasion, and SHP2 KD reversed the effects of SHP2 OE. mithramycin A promoted SHP2 KD tumor cell migration and invasion, and JQ-1 significantly promoted SHP2 KD tumor cell migration and invasion (Figures 5B,C). In summary, SHP2 in tumor-associated macrophages promotes the survival of gastric adenocarcinoma cells by inhibiting the P38/ERK1/2/SP1/BRD4/Sting-mediated inflammatory response and ROS production (Figure 6). The raw traces and raw data from this study were provided in Supplementary files 2, 3, respectively.

## 4 Discussion

In this study, we systematically investigated the roles of SHP2, SP1 and BRD4 in tumor-associated macrophages (TAMs) and their effects on apoptosis, migration, invasion and inflammatory responses of gastric adenocarcinoma cells. Based on the Bulk RNA-seq analysis of the GSE118916 dataset, we first found that the differentially expressed genes in gastric cancer tissues were significantly enriched in biological processes such as “extracellular matrix organization” and “wound healing.” The KEGG pathways were enriched in “ECM-receptor interaction” and “adhesion spot” pathways, suggesting that the tissue remodeling and cell adhesion functions in the tumor microenvironment play an important role in the development of gastric cancer. The immune infiltration analysis further revealed that regulatory T cells, Th2 cells, and activated CD4 + T cells dominated in gastric cancer tissues, and macrophages were significantly positively correlated with activated dendritic cells, suggesting their synergistic role in constructing an immunosuppressive microenvironment. The findings demonstrated that under hypoxic low-serum low-glucose circumstances, SHP2 expression was considerably downregulated in TAMs, whereas P38, ERK1/2, SP1, and BRD4 expression was significantly increased (22). Cancer cell-expressed CD47 interacted with the inhibitory receptor SIRPalpha on TAMs and recruited the protein tyrosine phosphatase SHP2. SHP2 recruitment not only suppressed the inflammatory response in TAMs, but also promoted the polarization of M2-type macrophages (23). M2-type macrophages provide favorable conditions for tumor growth and metastasis by secreting anti-inflammatory factors (e.g., IL-10 and TGF-*β*) and pro-angiogenic factors (e.g., VEGF) (24). In addition, activation of the CD47-SIRPα-SHP2 pathway inhibits phagocytosis by immune cells, thus enabling tumor cells to evade immune surveillance.

Recent research has demonstrated that TAMs, the most prevalent immune cell population in the tumor microenvironment, have a direct impact on the effectiveness of immune checkpoint inhibitors. Adenocarcinoma cells recruited SHP2 and encouraged TAM M2 polarization, which accelerated the growth of the tumor (25–27). For example, M2-type TAMs are strongly associated with immune treatment resistance because they create an immunosuppressive milieu that weakens T cells’ anti-tumor response by secreting anti-inflammatory molecules including TGF-β and IL-10 (28, 29). Additionally, inflammatory signals in the TME and oxidative stress status also participate in the regulation of immune therapy sensitivity (30). Interestingly, SHP2 showed significant differences in its role in TAMs and tumor cells. In tumor cells, activation of SHP2 promoted the expression of P38 and ERK1/2, two kinases that further regulate downstream inflammatory and proliferative signaling pathways by modulating the nuclear localization of SP1 and BRD4 (31, 32). In contrast, in TAMs, SHP2 reduces the nuclear localization of SP1 and BRD4 by inhibiting the expression of P38 and ERK1/2, thereby reducing the expression of inflammatory factors (33–35). This regulatory mechanism highlights the versatility and complexity of SHP2 in different cell types.

Important members of the MAPK family, P38 and ERK1/2, are crucial for cell division, proliferation, and death (36). By attaching to the GC box on DNA, SP1 functions as a transcription factor that controls the expression of many genes, including P2X7R (37). P2X7R is an ATP-gated ion channel that plays an important role in immune response and cell proliferation (38). In addition, BRD4 promotes the expression of PLK1, a serine/threonine kinase that promotes the phosphorylation of FOXM1, thereby inducing the polarization of tumor-associated macrophages to facilitate immune escape and metastasis, by recognizing acetylated histones (39). FOXM1 is an important cell cycle regulator, and its phosphorylation status determines cell proliferation and apoptosis (40). In this study, p-FOXM1 further activated downstream inflammatory and oxidative stress signaling pathways by promoting the synthesis of STING (41). p-FOXM1 is a key sensor in the innate immune system that senses intracellular DNA and induces the expression of inflammatory factors through the activation of TBK1 and IRF3 (42, 43). In the current study, hypoxic low serum low glucose conditions in advanced gastric adenocarcinoma stimulated the release of cGAMP, which binds to P2X7R and STING and activates TBK1 and IRF3. This in turn stimulated the expression of NLRP3, an inflammatory vesicle that stimulates the production of ROS and the release of inflammatory factors like TNFα and IL-1β (44).

In advanced gastric adenocarcinoma, the microenvironment of hypoxia, low serum and low glucose significantly enhances inflammation and oxidative stress. This microenvironment not only promoted the expression of NLRP3, but also increased the production of IL-1β, TNFα, and MDA, while decreasing the expression of SOD. MDA is a key product of lipid peroxidation, and its high level reflects the intensification of intracellular oxidative stress. And the reduced expression of SOD, an endogenous antioxidant, further exacerbated oxidative stress. The current study’s findings demonstrated that while overexpression of SHP2 reduced oxidative stress, knockdown of SHP2 markedly increased it, indicating a crucial role for SHP2 in controlling oxidative stress.

As transcriptional regulators, SP1 and BRD4 are crucial for controlling oxidative stress and inflammation. SP1 activates the STING signaling pathway by promoting the expression of P2X7R, which promotes the production of inflammatory factors (37, 45). BRD4, on the other hand, regulated the phosphorylation of FOXM1 by promoting the expression of PLK1, which further promoted the synthesis of STING and the activation of inflammatory responses (46). In the present study, the SP1 inhibitor Mithramycin A and the BRD4 inhibitor JQ-1 significantly suppressed the expression of inflammatory factors and oxidative stress, suggesting that SP1 and BRD4 are potential targets for the treatment of gastric adenocarcinoma.

This study also investigated the effects of SHP2, SP1 and BRD4 on apoptosis, migration and invasion of gastric adenocarcinoma cells. The results showed that apoptosis was significantly induced by hypoxic low-serum low-glucose conditions, whereas the overexpression of SHP2 inhibited apoptosis and promoted the survival of tumor cells. In contrast, knockdown of SHP2 significantly enhanced apoptosis. In addition, treatment with Mithramycin A and JQ-1 significantly inhibited apoptosis in SHP2 knockdown tumor cells, suggesting an important role of SP1 and BRD4 in the regulation of apoptosis. In terms of migration and invasion, overexpression of SHP2 significantly promoted the migration and invasion of tumor cells, whereas SHP2 knockdown inhibited these processes. The migration and invasion of SHP2 knockdown tumor cells were further enhanced by treatment with Mithramycin A and JQ-1, indicating a crucial role for SP1 and BRD4 in controlling cell migration and invasion. By releasing pro-inflammatory factors (like IL-12 and IFN-*γ*) and reactive oxygen species (ROS) that boosted the immune response, M1-type macrophages inhibited the growth and metastasis of tumors. In contrast, overexpression of SHP2 promoted M2 polarization in TAMs, which further promoted tumor development.

In summary, SHP2 in TAMs suppressed inflammatory responses and oxidative stress by inhibiting the P38/ERK1/2/SP1/BRD4/STING signaling pathway, thereby promoting gastric adenocarcinoma survival, migration, and invasion. Activation of SHP2 promoted the M2 polarization of TAMs, whereas inhibition of SHP2 promoted the M1 polarization of TAMs, further inhibiting tumor progression. Furthermore, SP1 and BRD4 are important regulators of oxidative stress and inflammation, and they may be used to treat gastric adenocarcinoma. The study’s findings offer fresh approaches to treating gastric adenocarcinoma. By interfering with the function of SHP2 in TAMs or inhibiting the expression of SP1 and BRD4, the progression of gastric adenocarcinoma may be effectively inhibited. Future studies could further explore the specific mechanisms of action of SHP2, SP1 and BRD4, as well as their potential applications in other types of tumors.

## Data Availability

The raw data supporting the conclusions of this article will be made available by the authors, without undue reservation.
