# Supplementary material for: SHP2 in TAMs promoted the survival of gastric adenocarcinoma via suppressing the P38/ERK1/2/SP1/BRD4/STING induced inflammation and ROS
Source: Front Med (Lausanne). 2026 Apr 30;13:1789222. doi: 10.3389/fmed.2026.1789222 (PMC13171397; doi:10.3389/fmed.2026.1789222)
Supplement: Supplementary file 1 [file Supplementary_File_1.docx]

Result:

The Spearman correlation analysis revealed that multiple key genes showed positive correlations, including CD47 with SIRPα, SIRPα with SHP2 (PTPN11), ERK1/2 (MAPK1) with Egr1, and Egr1 with BRD4, suggesting that these genes may interact with each other through a complex signaling network to jointly regulate the biological characteristics of gastric adenocarcinoma; among them, NLRP3 was positively correlated with the inflammatory marker IL-6, indicating that NLRP3 may play an important role in the inflammatory response of gastric adenocarcinoma.


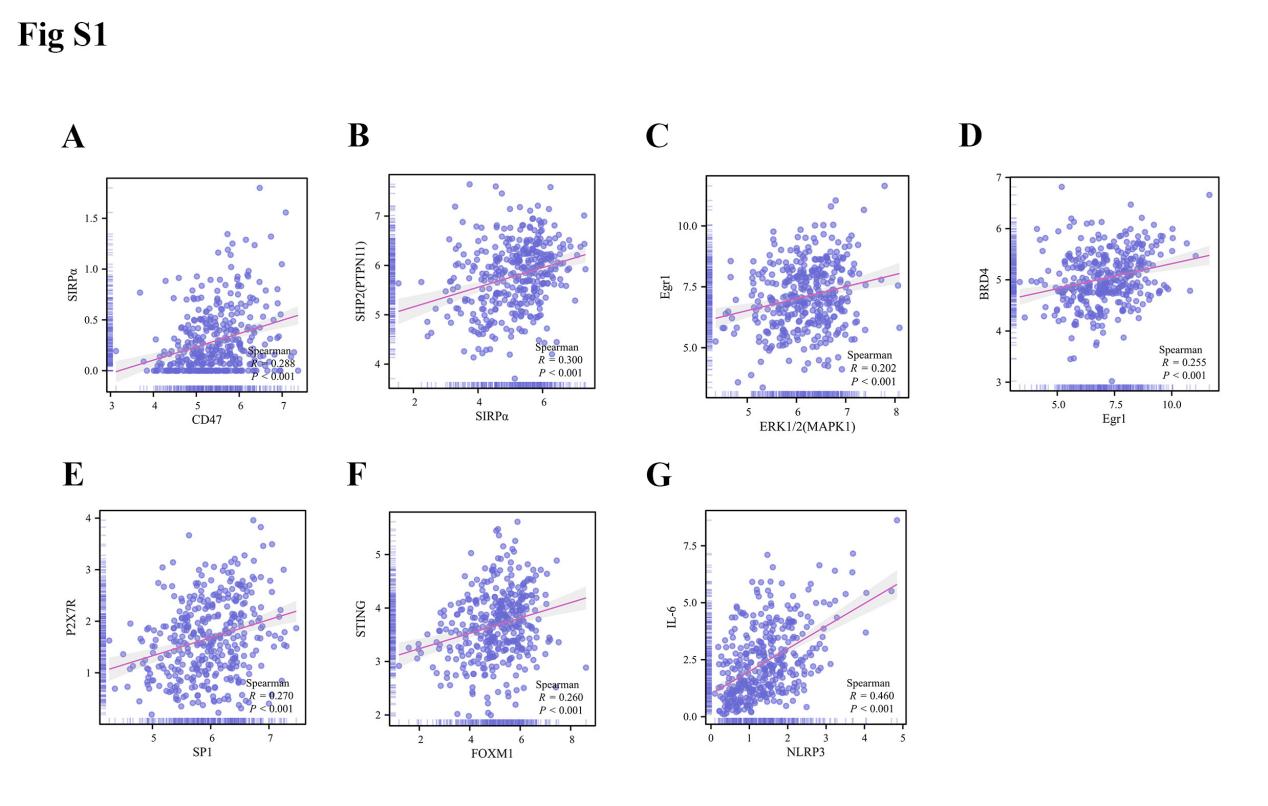


Fig S1: Results of Spearman's correlation analysis

A. Scatter plot of the correlation between CD47 and SIRPα; B. Scatter plot of the correlation between SIRPα and SHP2 (PTPN11); C. Scatter plot of the correlation between ERK1/2 (MAPK1) and Egr1; D. Scatter plot of the correlation between Egr1 and BRD4; E. Scatter plot of the correlation between SP1 and P2X7R; F. Scatter plot of the correlation between FOXM1 and STING; G. Scatter plot of the correlation between NLRP3 and IL-6.
