# Supplementary figures and images for: SHP2 in TAMs promoted the survival of gastric adenocarcinoma via suppressing the P38/ERK1/2/SP1/BRD4/STING induced inflammation and ROS
Source: Front Med (Lausanne). 2026 Apr 30;13:1789222. doi: 10.3389/fmed.2026.1789222 (PMC13171397; doi:10.3389/fmed.2026.1789222)

Supplementary File 2

Fig2A

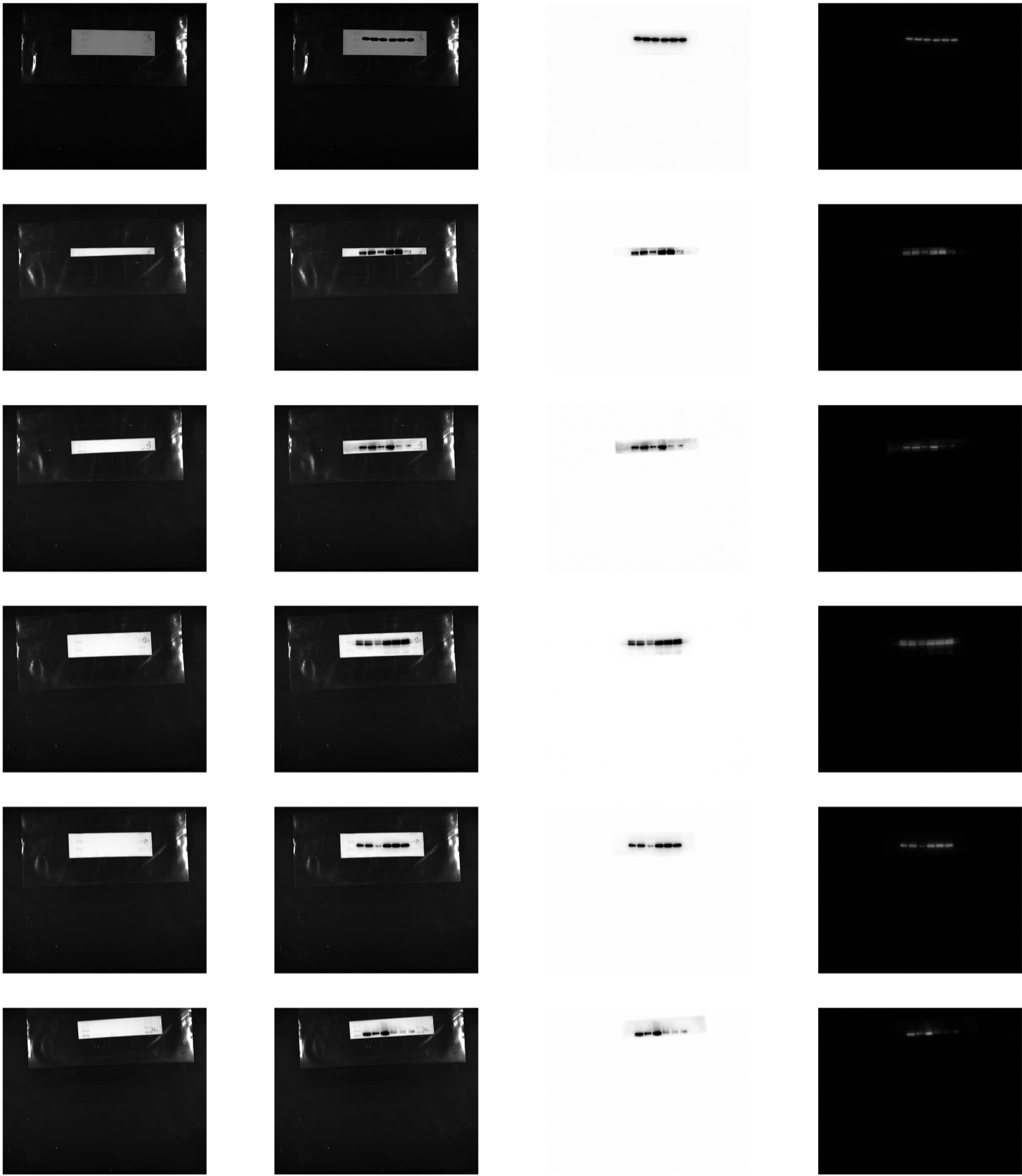

Fig2B

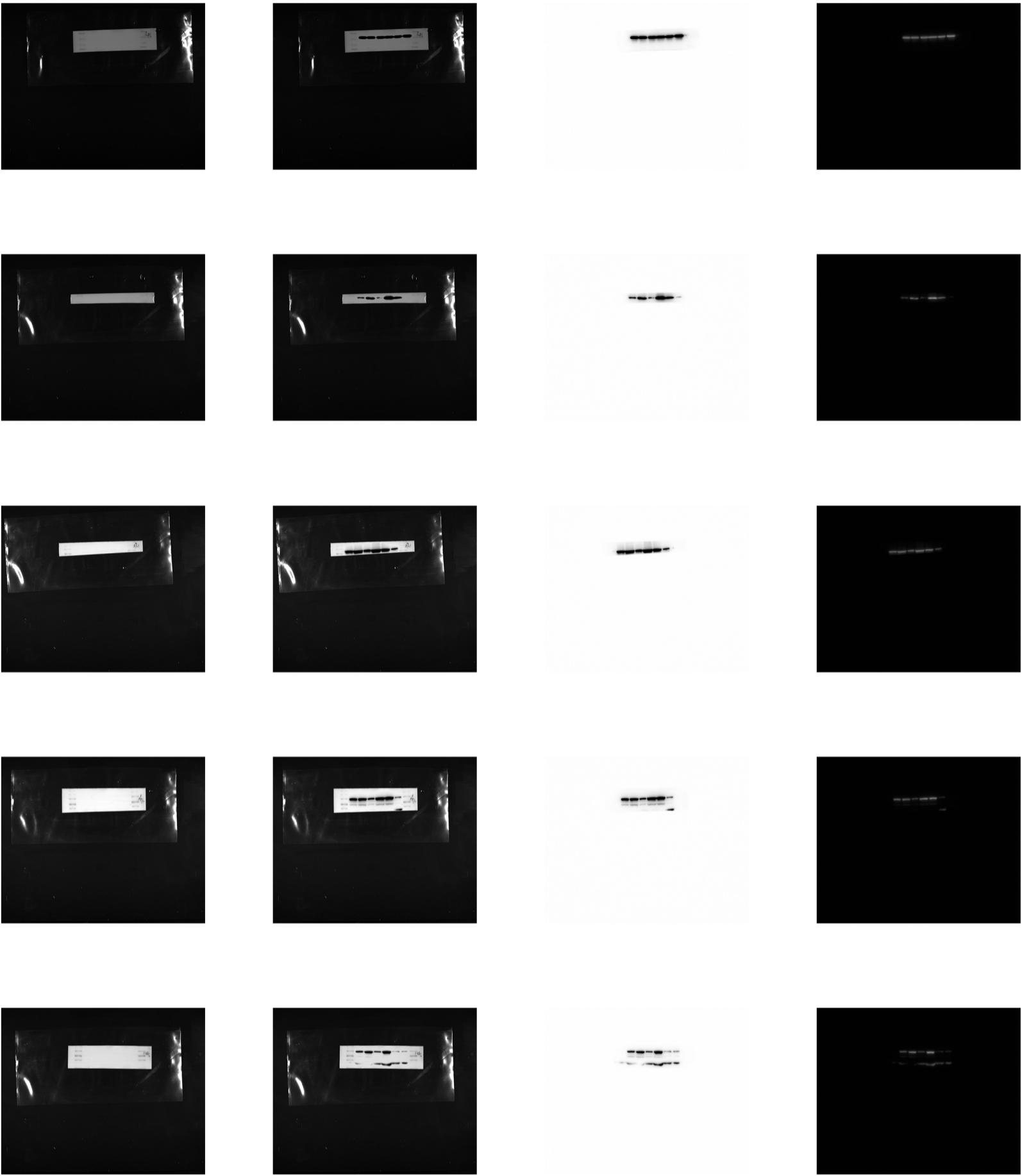

P2X7R

p-FOXM1

STING

NLRP3

GAPDH

Fig4A

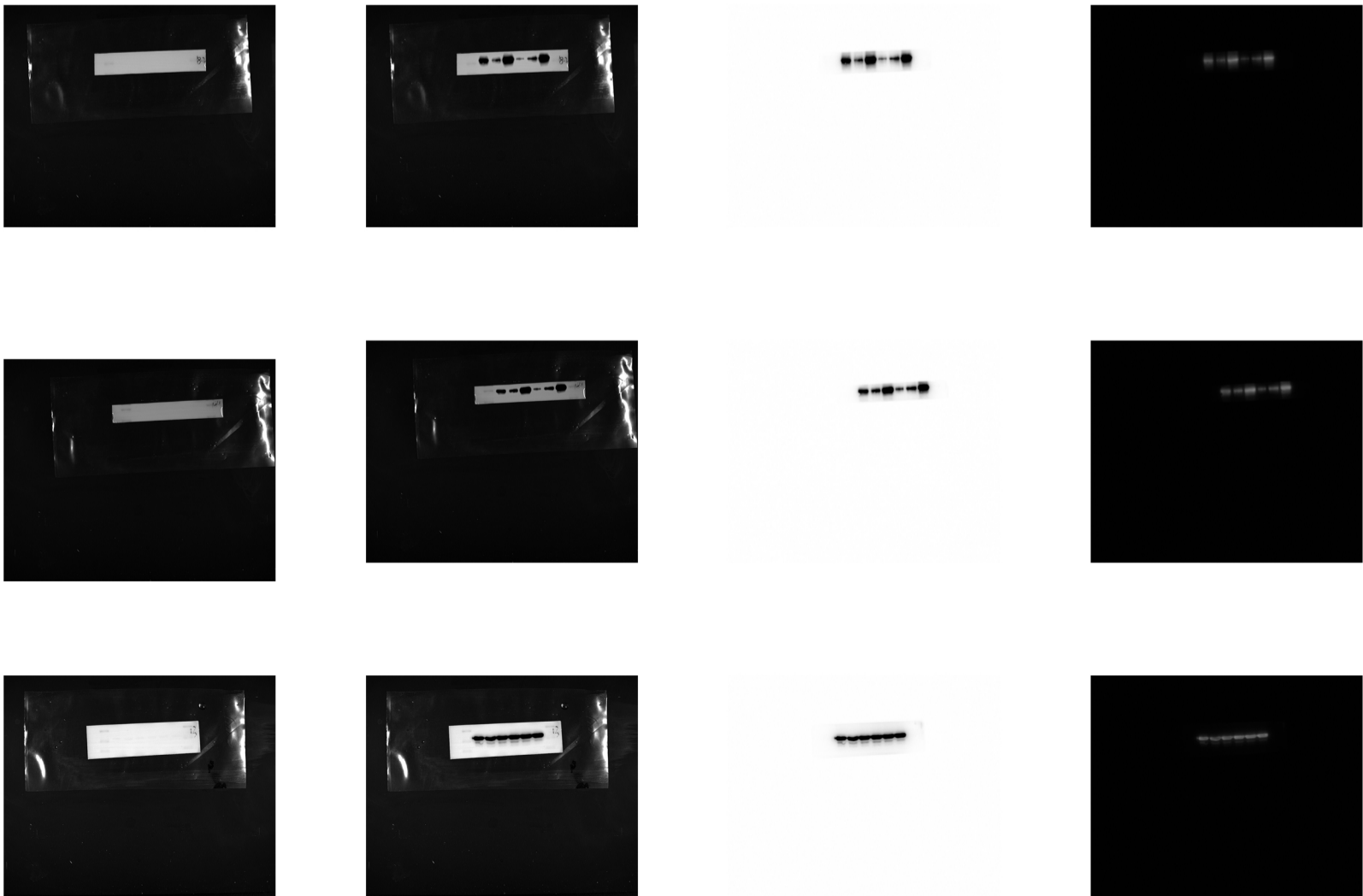

arginase-1

Cathepsin

GAPDH

Supplement: Supplementary file 2 [file Supplementary_File_2.pdf]
